# Rare Case of Bilateral Diffuse Uveal Melanocytic Proliferation with Dermal and Mucosal Hyperpigmentations

**DOI:** 10.3390/diagnostics11112052

**Published:** 2021-11-05

**Authors:** Michelle Prasuhn, Nathalie Rommel, Vinodh Kakkassery, Salvatore Grisanti, Mahdy Ranjbar, Felix Rommel

**Affiliations:** 1 Department of Ophthalmology, University of Lübeck, University Hospital Schleswig-Holstein, Ratzeburger Allee 160, 23538 Lübeck, Germany; Vinodh.Kakkassery@uksh.de (V.K.); Salvatore.Grisanti@uksh.de (S.G.); eye.research101@gmail.com (M.R.); felix.rommel@uksh.de (F.R.); 2 Department of Dermatology, University of Lübeck, University Hospital Schleswig-Holstein, Ratzeburger Allee 160, 23538 Lübeck, Germany; nathalie.rommel@uksh.de

**Keywords:** bilateral diffuse uveal melanocytic proliferation, dermal hyperpigmentation, mucosal hyperpigmentation, serous retinal detachment, leopard spot chorioretinitis, lung carcinoma, fundus autofluorescence, OCT, paraneoplastic syndrome

## Abstract

Purpose: The demonstration of a rare case of bilateral diffuse uveal melanocytic proliferation (BDUMP) due to a lung carcinoma with unusual dermal lesions. Case description: A 76-year-old man with painless bilateral vision loss revealed leopard or giraffe spot chorioretinopathy and bilateral serous retinal detachment. Ultrasound biomicroscopy revealed uveal swelling expanding into the anterior chamber angle. Dermal and mucosal lesions were present on the lip, breast, groin, scrotum, and penis. Screening analyses revealed a non-small cell lung carcinoma. Conclusions: The diagnosis of BDUMP, a rare paraneoplastic syndrome, was made. The ophthalmological diagnosis led to screening investigations and revealed the underlying malignant disease. Uncommonly, multiple dermal and mucosal lesions could be detected and were analyzed histopathologically.

## 1. Introduction

Paraneoplastic syndromes are a group of rare disorders caused by certain neoplasms that trigger erroneous attacks of the immune system. Bilateral diffuse uveal melanocytic proliferation (BDUMP) is one of these rare conditions and is characterized by a rapid proliferation of intraocular melanocytes into the uvea, typically leading to multiple, elevated pigmented chorioretinal lesions and exudative retinal detachments.

Here, we present the case of a patient with BDUMP and unusual dermal lesions in the context of a non-small cell lung carcinoma.

## 2. Case Description

**Figure 1:**
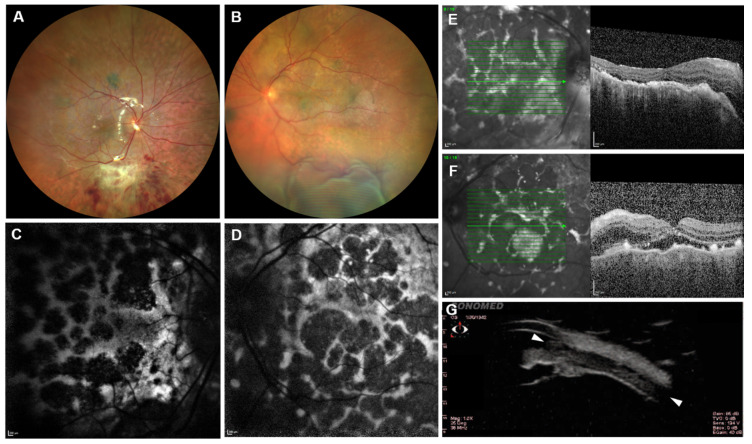
Ophthalmological findings. (**A**): Fundus image of the right eye at presentation, after vitrectomy and silicone oil filling. Diffuse flat, dark spots are distributed over the posterior pole. (**B**): Vitreous haze and retinal detachment in the left eye, also showing dark spots. (**C**,**D**): Fundus autofluorescence ((**C**): right eye; (**D**): left eye) with a leopard spot pattern. (**E**,**F**): OCT B-scans ((**E**): right eye; (**F**): left eye) reveal subretinal fluid, exudates, and distorted retinal layers. (**G**): Ultrasound biomicroscopy showing uveal swelling expanding into the anterior chamber angle (arrowhead to arrowhead).

A 76-year-old man was referred to our clinic with a decrease in visual acuity due to a retinal detachment on his left eye. Six weeks earlier he had been treated with pars plana vitrectomy and silicone oil tamponade for a retinal detachment on his right eye in an external clinic. According to the surgical report, a drainage retinotomy was created to drain the subretinal fluid, since no retinal breaks were detected. The patient reported of uncomplicated cataract surgeries on both eyes in the past, and further ophthalmological and general medical history were unremarkable. Best corrected visual acuity (BCVA) was 20/80 on the right eye and 20/200 on the left with normal intraocular pressures. Slit lamp examination of both anterior segments revealed a slight chemosis but no other pathologies. Fundus examination of the right eye revealed few retinal hemorrhages on an attached retina with silicone oil tamponade (Figure 1A). The left eye had a modest vitreous haze with serous retinal detachment and a protruding uveal swelling extending into the anterior chamber angle measuring 8.2 mm × 1.3 mm on ultrasound biomicroscopy (Figure 1B,G). Moreover, some flat, small, and diffuse naevus-like melanotic changes of the retina were noticed on both eyes, right more than left. Optic coherence tomography (OCT) revealed a macular edema with subretinal fluid and hard exudates (Figure 1E,F). Fundus autofluorescence (AF) revealed giraffe or leopard spots with intervening areas of increasing and decreasing AF (Figure 1C,D).

**Figure 2:**
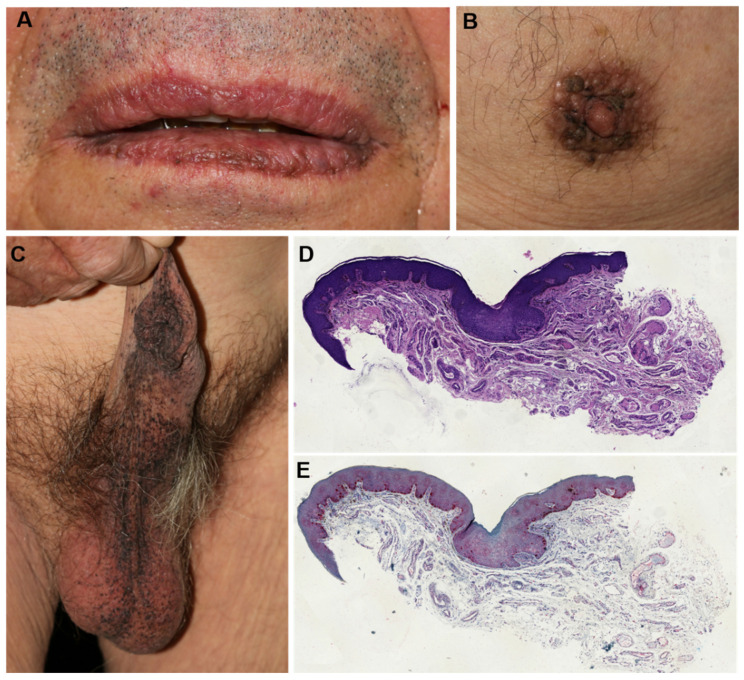
Dermatological examination and histopathological findings. Dermal and mucosal pigmentations of the (**A**): mouth, (**B**): nipple–areola, (**C**): penis and scrotum. (**D**): Hematoxylin and eosin stain of a lip biopsy shows acanthotic epithelium with basal hyperpigmentation and focally increased melanophages in the superficial dermis (medium magnification). (**E**): Immunohistochemical stain with Melan-A reveals junctionally regularly distributed melanocytes that are enlarged but not ascending (medium magnification).

Upon these findings, we carried out a more specific medical history, which revealed no known neoplasms but a weight loss of 10 kg over the last months, 30 pack years, and dark dermal spots of the mouth, breast, groin, scrotum, and penis that developed over the past eight weeks (Figure 2A–C). With the suspected diagnosis of BDUMP, we initiated screening investigations, including computed tomography of the thorax and abdomen, and dermatological co-evaluation of the skin lesions. A biopsy of the hyperpigmentation of the lip was taken and revealed an acanthotic epithelium with basal hyperpigmentation and focal proliferation of melanophages but without evidence of malignancy, compatible with the diagnosis of a lentigo (Figure 2D,E; for higher resolution images and greater magnification see Figures S1–S4).

On thoracic computed tomography, subpleural consolidation and apical lesions of both lungs, as well as swollen hilar lymph nodes suspicious for metastasis, were unmasked (Figure S5). Apart from the aforementioned unintentional weight loss and consumption of cigarettes, other clinical signs suggestive of lung carcinoma such as chronic cough, dyspnea, and hemoptysis were not present. The patient was referred to our pulmonary clinic, in which a transbronchial biopsy was taken. The diagnosis of non-small cell lung carcinoma (NSCLC) with squamous cell histology was made (immunohistochemistry: PD-L1 20% positive, CK 5/6 and p40 positive; mutation status: KRAS and BRAF wild type). Further imaging staging analyses with abdominal CT, MRI of the head, and PET-CT showed no further lesions, leading to the TNM stage cT2a cN2 cM1a.

The patient underwent atypical segmentectomy via video-assisted thoracic surgery, and adjuvant chemotherapy with carboplatin and vinorelbine was initiated. After therapy initiation, the patient decided not to be followed up at our pulmonary clinic, and refused any further treatment.

## 3. Discussion

Paraneoplastic syndromes are complexes of signs and symptoms resulting from the dysfunction of tissues remote from the site of a malignant neoplasm or its metastases [1]. BDUMP is a rare intraocular paraneoplastic syndrome due to extraocular neoplasms with uveal proliferations that are histopathologically unrelated to the underlying tumor. 

Gass et al. characterized this condition with five typical findings: (I) multiple, subtle, round or oval red patches in the fundus; (II) multifocal early hyperfluorescence corresponding with these patches; (III) diffuse thickening of the uveal tract with elevated focal tumors; (IV) exudative retinal detachment; (V) rapid progression of cataracts [2]. As our patient was already pseudophakic, he met all but one criterion on both eyes. To the best of our knowledge, this is the first case report demonstrating uveal thickening extending beneath the ciliary body to the anterior chamber angle by using ultrasound biomicroscopy in a BDUMP patient. Moreover, BDUMP can be associated with multifocal dermal and mucosal hyperpigmentations, the latter occurring more often than the former [3]. However, case reports on these patients and histological findings are rare. In the few cases reported, only single hyperpigmentations were described without performing histological examination. Singh et al. suggested to sum this phenomenon up as “Paraneoplastic melanocytic proliferation”, whereas Pulido et al. would rather term it “bilateral diffuse uveal and focal dermal melanocytic proliferation (BDUFDMP)” [3,4]. Pigmented lesions usually show hyperpigmentation of the basal cell layer, irregular elongation of the rete ridges, and scattered melanocytes and melanophages in the underlying submucosa or dermis. Nodular proliferation of melanocytic cells in the mid-dermis has also been documented [3,5,6,7].

In about half of BDUMP cases, the ophthalmological diagnosis is made before an underlying malignancy is detected [8]. In our case, the patient noticed unintentional weight loss and the aforementioned hyperpigmentations. However, these symptoms did not make him seek medical assistance, but the vision loss did. Other symptoms suggestive of pulmonary malignancies were not present. Therefore, it is crucial for ophthalmologists to include systemic diseases and especially paraneoplastic syndromes into their differential diagnostic thinking as they can be the first doctors a patient consults.

Women are predominantly affected, and the condition is often triggered by primarily endocrine urogenital carcinomas [8]. As demonstrated in our case, half of the male patients appear to have a pulmonary carcinoma [8]. Case reports of BDUMP in the past often did not differ between small cell and non-small cell carcinoma. Small cell lung carcinomas are usually more active when it comes to endocrine or paraneoplastic activity, so it can be assumed that BDUMP is also more often triggered by this entity. However, a biopsy in our patient verified the diagnosis of non-small cell lung carcinoma (immunohistochemistry: PD-L1 20% positive, CK 5/6 and p40 positive).

The exact pathomechanisms of BDUMP are not fully understood, mainly due to its rarity. However, progress was made in the last decade as a substance of the IgG fraction could be identified that was extracted from BDUMP patients. This cultured melanocyte elongation and proliferation (CMEP) factor is hypothesized to be either secreted by the cancer cells or produced by the immune system in response to the cancer and causes cultured melanocytes to proliferate [9,10].

Therapy of BDUMP can be difficult, even when the correct diagnosis is made. Data of the last decade showed that the findings can be ameliorated by plasmapheresis and plasma exchange [11]. However, patients diagnosed with BDUMP have an overall mean survival of 15.7 months after presentation [8]. This case was presented in our local multidisciplinary tumor board, which is an important step for therapy initiation in non-small cell lung cancer. After atypical segmentectomy, our patient received adjuvant chemotherapy with carboplatin and vinorelbine, which is in accordance with the current guidelines in Germany [12]. Targeted immunotherapy was not started. After the initial therapy, our patient refused any further treatment, and unfortunately decided not to be followed up in our ophthalmological or dermatological clinic.

## 4. Conclusions

In conclusion, the presented case highlights the importance of accurate diagnostics in patients presenting with leopard or giraffe spot chorioretinopathy and serous retinal detachment for the diagnosis of BDUMP. The role of the ophthalmologists and their differential diagnostic thinking is crucial, as the carcinoma itself may be asymptomatic for a long time, which hampers an early diagnosis and therapy induction. Besides typical findings on slit lamp examination, OCT and AF, ultrasound biomicroscopy can be helpful to estimate the expansion of uveal effusion. Moreover, patients should be examined for cutaneous and mucosal pigmentation and an interdisciplinary screening for underlying malignancy should be initiated.

## Supplementary Materials

The following are available online at https://www.mdpi.com/article/10.3390/diagnostics11112052/s1, Figure S1: biopsy of the lip: high resolution image of the hematoxylin and eosin stain; Figure S2: biopsy of the lip: high resolution image of the Melan-A stain; Figure S3: higher magnification of the hematoxylin and eosin stain of the lip biopsy; Figure S4: higher magnification of the Melan-A stain of the lip biopsy; Figure S5: CT-scan of the chest revealing the lung carcinoma.
