# Supplementary figures and images for: Rare Case of Bilateral Diffuse Uveal Melanocytic Proliferation with Dermal and Mucosal Hyperpigmentations
Source: Diagnostics (Basel). 2021 Nov 5;11(11):2052. doi: 10.3390/diagnostics11112052 (PMC8619430; doi:10.3390/diagnostics11112052)

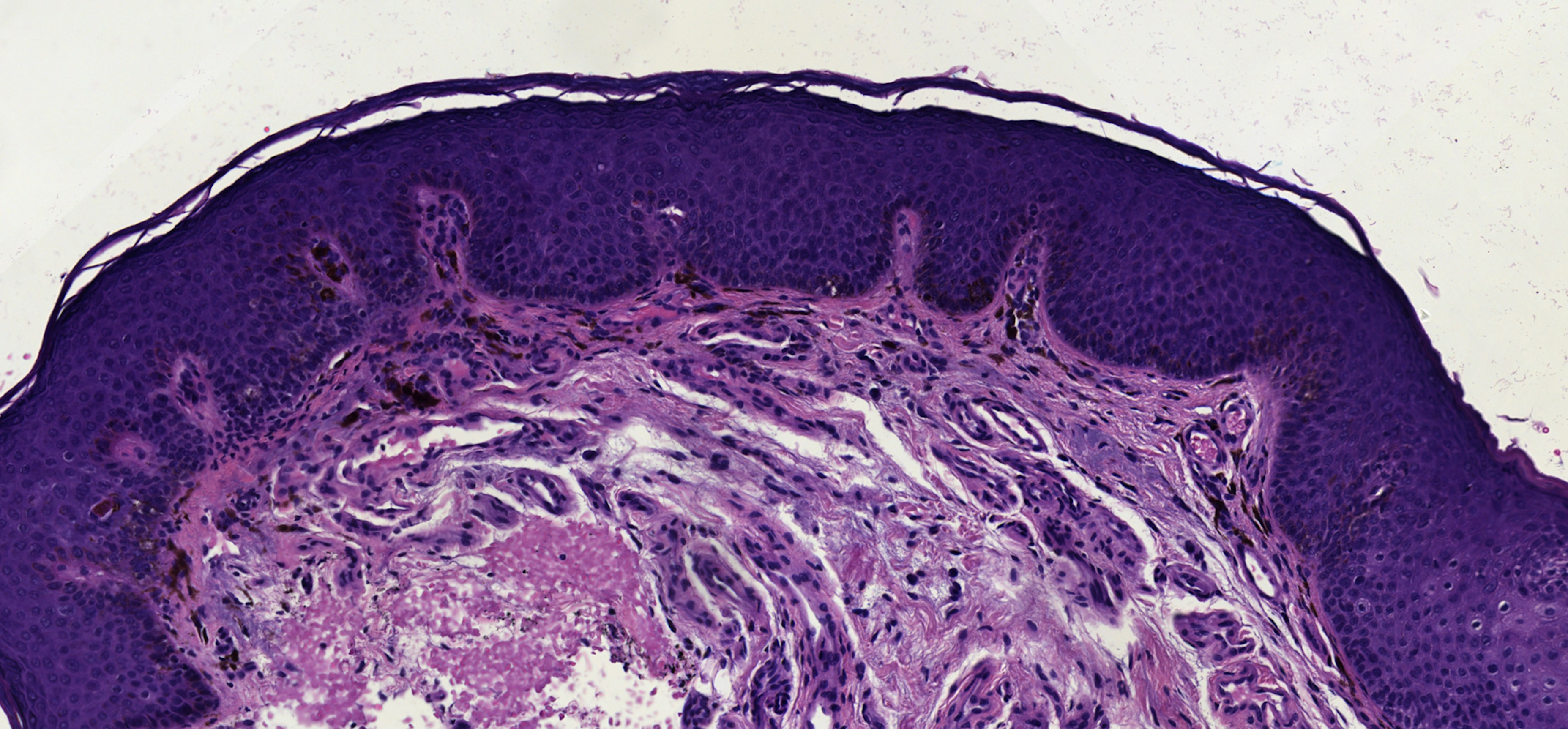

Supplement: Supplementary file 1 [file diagnostics-11-02052-s001.zip › diagnostics-1405655-supplementary/Supplem_new/Figure S3.tiff]

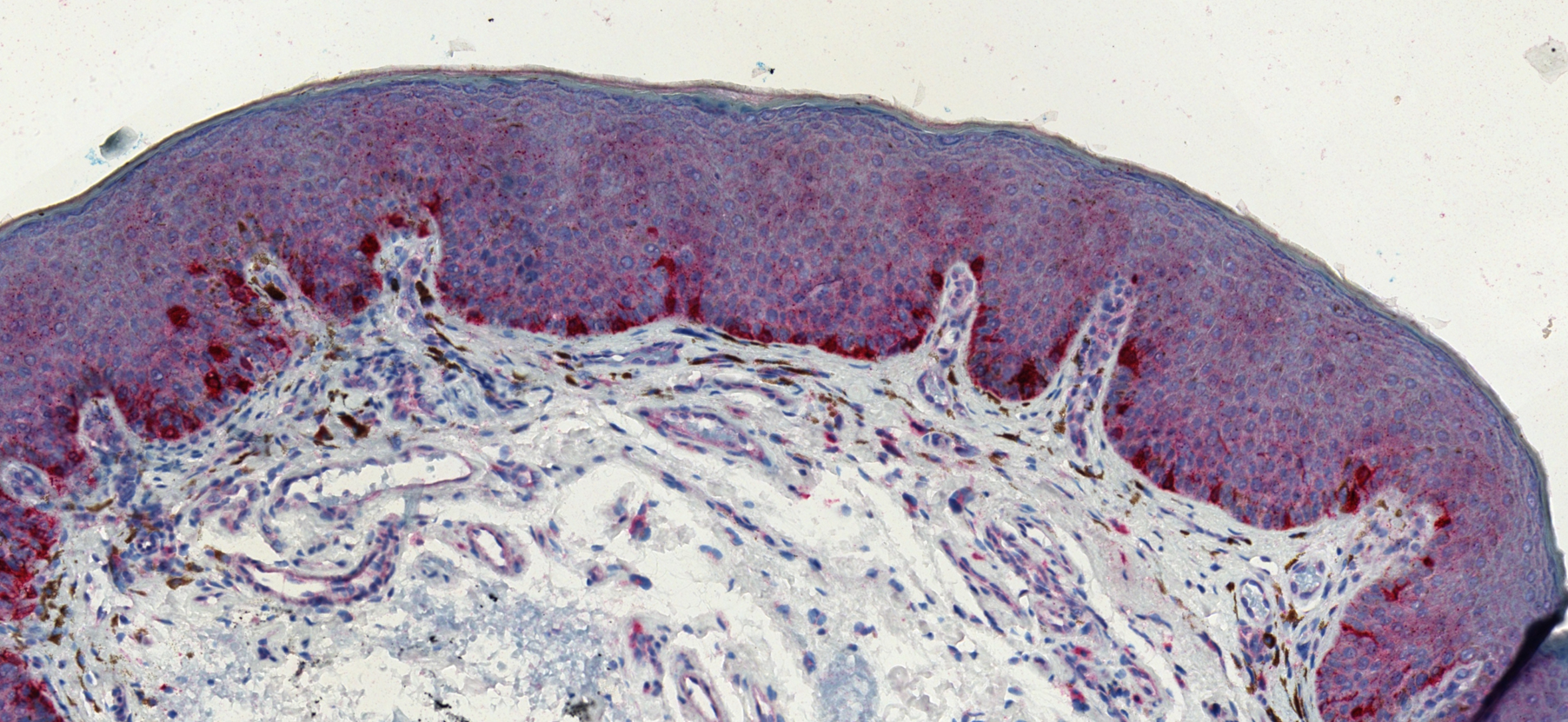

Supplement: Supplementary file 1 [file diagnostics-11-02052-s001.zip › diagnostics-1405655-supplementary/Supplem_new/Figure S4.tiff]

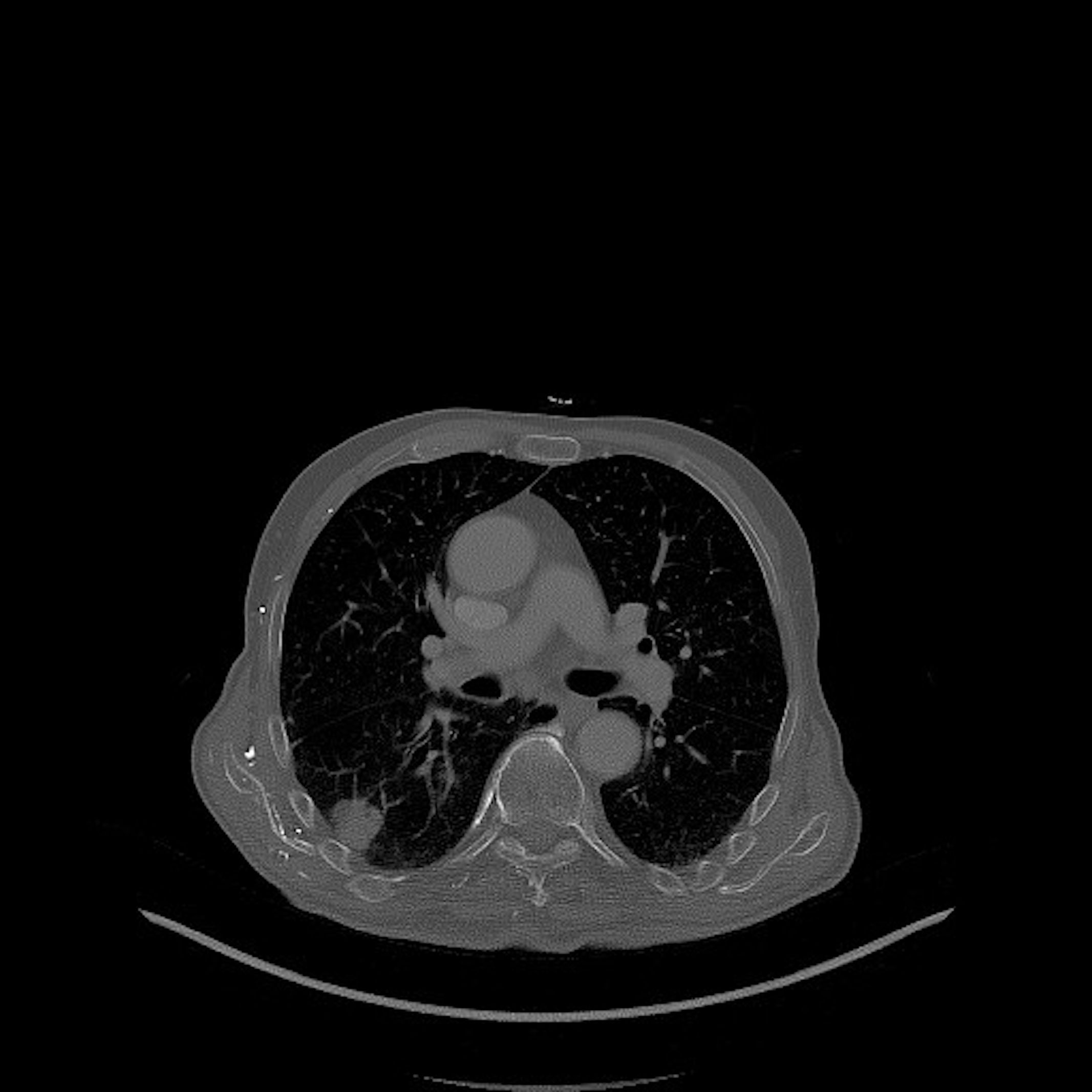

Supplement: Supplementary file 1 [file diagnostics-11-02052-s001.zip › diagnostics-1405655-supplementary/Supplem_new/Figure S5.tiff]
